# Spinal Cord Medulloepithelioma in a Cat

**DOI:** 10.3390/vetsci11040177

**Published:** 2024-04-15

**Authors:** Çağla Aytaş, Raffaele Gilardini, Annalisa Beghelli, Paolo Andrea Barili, Melissa Ori, Carlo Cantile

**Affiliations:** 1 Department of Veterinary Science, University of Pisa, 56126 Pisa, Italy; cagla.aytas@phd.unipi.it; 2 Clinica Veterinaria Città di Voghera, 27058 Voghera, Italy; raffagilardini@gmail.com (R.G.); beghe.beghelli@gmail.com (A.B.); paoloandreabarili@gmail.com (P.A.B.); mela.ori89@gmail.com (M.O.)

**Keywords:** cat, central nervous system tumors, immunohistochemistry, primitive neuroectodermal tumor, medulloepithelioma

## Abstract

**Simple Summary:**

A primitive neuroepithelial tumor with the morphological features of medulloepithelioma is described in a young cat with progressive paraparesis. Magnetic resonance showed a dorsal intradural extramedullary space-occupying lesion extending from L5 to L6. The histomorphology and immunoreactivity of the neoplastic cells for vimentin and NSE resembled human medulloepithelioma. This is the first spinal cord medulloepithelioma report in a cat.

**Abstract:**

A 13-month-old, neutered, male, domestic shorthair cat was referred with a history of progressive paraparesis, proprioceptive ataxia, and lumbar spinal pain. Neurological examination revealed non-ambulatory paraparesis consistent with L4-S1 myelopathy. Magnetic resonance of the thoracolumbar spinal cord identified a dorsal intradural extramedullary space-occupying lesion extending from L5 to L6. It was homogeneously hyperintense in T2-weighted imaging and isointense in T1-weighted imaging and exhibited marked and homogeneous contrast enhancement in the T1-weighted post-contrast imaging. The removed tissue was composed of neoplastic cells arranged as pseudostratified or multilayered trabecular and tubular structures, supported by internal and external limiting PAS-positive membranes. The neoplastic cells were immunoreactive for vimentin and NSE and negative for GFAP, Olig2, synaptophysin, PCK, S-100, NeuN, and nestin. The Ki-67 nuclear labeling index was up to 90%. The tumor was consistent with the diagnosis of medulloepithelioma, which is most frequently reported as an intraocular tumor. The morphological and immunohistochemical features of the tumor showed remarkable concordance with most human medulloepitheliomas. This is the first spinal cord medullopethelioma report in a cat, with the clinical, neuroradiological, histological, and immunohistochemical findings being described.

## 1. Introduction

Medulloepithelioma (MEPL) is a rare, highly malignant primitive neuroectodermal tumor characterized by tubular and trabecular arrangements of tumor cells. In animals, MEPL has been mostly described as an intraocular tumor in horses [1,2,3,4], dogs [5,6,7,8,9], cats [10,11], llamas [12,13], and cockatiels [14,15], with single case descriptions in a goldfish [16], an African grey parrot [17], and a northern, red-shouldered macaw [18]. A primary intraocular neuroepithelial embryonal tumor with histological features of retinoblastoma and MEPL has been reported in two rabbits [19]. Metastases of intraocular MEPL to the brain and kidneys have been described in a dog [8] and to the lymph nodes, lungs, and liver in a horse [3] and llama [13]. Primary extraocular MEPLs in the canine spinal cord have been reported in the earlier veterinary literature [20,21,22]. However, it is currently believed that the ectopic nephroblastoma of young dogs has historically been misdiagnosed as MEPL and that this tumor variant has not yet been recognized in the central nervous system (CNS) of domestic animals.

In humans, MEPL is more common in children and young adults, typically arising in the periventricular regions of the brain [23,24,25], and as an intraocular tumor from the ciliary body [26]. Exceptional locations include the spinal cord [27,28,29] and the optic [27] and sciatic nerve [30].

In this report, we describe the clinical, neuroradiological, and pathological features of a primary spinal cord MEPL in a young cat.

## 2. Case Description

A 13-month-old, neutered, male, domestic shorthaired cat was referred with a clinical history of progressive paraparesis, proprioceptive ataxia, and lumbar spinal pain. Two months earlier, the cat showed difficulty in jumping and spinal pain that was treated with meloxicam (0.1 mg/kg q24h orally for 7 days) with partial response. Plantigrade stance and proprioceptive ataxia were noticed approximately one month following the onset of symptoms. At referral, neurological examination revealed non-ambulatory paraparesis and depressed patellar and withdrawal reflexes in both pelvic limbs. The trunk cutaneous reflex was absent caudally from L3, and the perineal reflex was normal. Lumbar hyperesthesia was noticed on palpation. These findings were deemed consistent with L4-S2 myelopathy.

**Figure 1:**
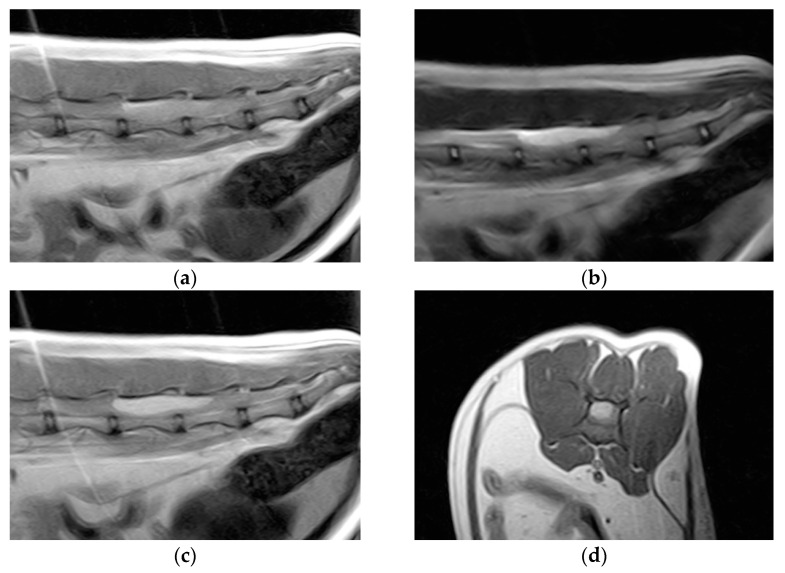
Magnetic resonance imaging of the spinal cord of the cat. (**a**) Preoperative sagittal T1-weighted image showing an isointense lesion on the dorsal side of the thoracolumbar spinal cord extending from L5 to L6. (**b**) The lesion is hyperintense on the T2-weighted images. (**c**) Marked lesion enhancement visualized on contrast-enhanced sagittal T1-weighted images. (**d**) Contrast-enhanced transverse T1-weighted image.

Hematology and blood biochemistry were unremarkable. Thoracolumbar column radiographs were normal. The cat was anesthetized and positioned for magnetic resonance imaging (MRI) of the thoracolumbar spinal cord (Esaote Vet-MR 0.2T, Genoa, Italy). T1-weighted sagittal and transverse scans, T2-weighted sagittal and transverse scans, and T1-weighted sagittal and transverse scans post-contrast (0.1 millimoles/kg of gadoteric acid—Claryciclic^®^, GE Healthcare s.r.l., Milano, Italy) were acquired. MRI identified an intradural extramedullary space-occupying mass extending from L5 to L6. The mass was dorsal and elliptical in shape (35 mm length, 8.3 mm width, and 7.2 mm height) and had sharp margins. It was homogeneously hyperintense in T2-weighted and isointense in T1-weighted scans and exhibited marked and homogeneous contrast enhancement in the T1-weighted post-contrast scans. The mass caused severe spinal cord compression (Figure 1).

A cerebrospinal fluid (CSF) sample obtained from the cisterna magna showed a cell count lower than 2 cells/µL (reference range < 5 cells/µL) consisting of cells from the monocytic series. The total proteins in the CSF were within the normal range (semiquantitative levels < 30 mg/dL on a urine dipstick). The owner agreed to the proposed surgical procedure, aimed at maximizing mass excision and providing tissue samples for histopathological analysis. The cat was positioned and stabilized in sternal recumbency. Perioperative cefazoline (20 mg/kg, q8h IV) was administered for antibiosis. After a midline incision over the lumbar vertebrae, a dorsal laminectomy was performed from the cranial limit of L4 to the end of L6 with a burr and small rongeurs. A left dorsolateral durotomy was then performed along the entire length of the exposed spinal cord, with the aim of exploring and removing the tumor. Small hemorrhages were controlled using absorbable hemostatic sponges. The dura mater was not sutured. Routine suture of the epaxial musculature, fascia, subcutis and dermis was performed.

**Figure 2:**
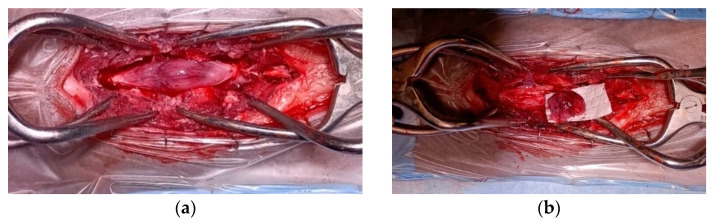
Spinal cord medulloepithelioma in the cat. (**a**) After durotomy, the appearance of the intradural-extramedullary space-occupying mass extending from L5 to L6; (**b**) the medulloepithelioma after resection.

Grossly, the tumor had a gelatinous and reddish appearance and was adherent but easily separable from the dura mater and infiltrated the spinal cord parenchyma (Figure 2). Using a surgical microscope, the mass was meticulously and gently removed by detaching it from the dura mater and the spinal cord parenchyma. However, complete removal could not be assured due to the difficulty in distinguishing the margins between the neoplastic tissue and the edematous spinal cord parenchyma.

Postoperative treatment included cefalexin 25 mg/kg q12h for 5 days and meloxicam 0.05 mg/kg q24h for 5 days. The owner refused other treatments. The cat improved rapidly and gradually regained autonomous ambulation and was discharged 3 days after surgery. At the 12-day post-surgery follow-up, ambulation was normal, and the cat’s clinical condition remained stable. Fourteen months post-surgery, the cat experienced difficulties in jumping, and a new MRI revealed tumor recurrence at the site of surgery. The owner declined any further treatment and chemotherapy. At the last follow-up, 1 month after MRI, the patient was alive and ambulatory with paraparesis.

The removed mass was fixed in 10% neutral buffered formalin solution. The tissue was routinely processed and embedded in paraffin, and 4 µm thick histological sections were stained with hematoxylin and eosin (H&E) and periodic acid–Schiff (PAS). Immunohistochemistry (IHC) was performed using the avidin–biotin–peroxidase complex method. Antigen retrieval was carried out using steam heat. Primary antibodies against the following antigens were used: vimentin (VIM; monoclonal, clone Vim 3B4, dilution 1:100; Dako, Carpinteria, CA, USA), neuronal specific enolase (NSE; monoclonal, clone NSE-P1, dilution 1:200; Sigma-Aldrich, St. Louis, MO, USA), nestin (monoclonal, clone Rat-401, dilution 1:500; Abcam, Boston, MA, USA), glial fibrillary acidic protein (GFAP; polyclonal, dilution 1:2000; Sigma-Aldrich), pancytokeratin (PCK; monoclonal, clone PCK-26, dilution 1:500; Sigma-Aldrich), Olig2 (polyclonal, dilution 1:500; Sigma-Aldrich), S-100 (polyclonal, dilution 1:200; Dako), NeuN (monoclonal, clone A60, dilution 1:500; Sigma-Aldrich), synaptophysin (monoclonal, clone SY38, dilution 1:500; Dako), and Ki-67 (monoclonal, clone MIB-1, dilution 1:250; Dako). The EnVision Plus System-HRP (3,3′-diaminobenzidine, Dako) was used to detect antibody binding. Negative controls were obtained by omitting the primary antibody.

**Figure 3:**
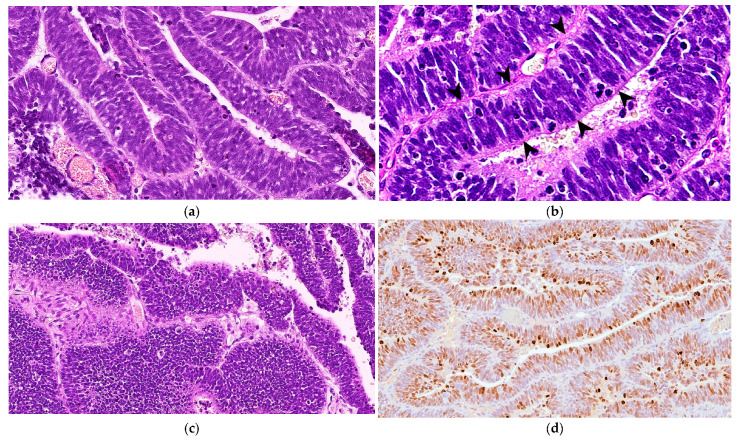
Spinal cord medulloepithelioma in the cat. (**a**) The tissue is formed by ribbons and trabecular aggregates of multilayered neoplastic neuroepithelium (H&E, 350×). (**b**) The tumor features PAS-positive basement membrane material along the external surfaces of the rosette and trabecular structures with barely identifiable limiting membranes in the internal surface (arrowheads) (PAS, 500×). (**c**) Occasionally, the tumor shows areas of poorly differentiated cells arranged in sheets (H&E, 200×). (**d**) Extensive nuclear immunoreaction for Ki-67 of almost all of the neoplastic cells (Ki-67 IHC, 175×).

**Figure 4:**
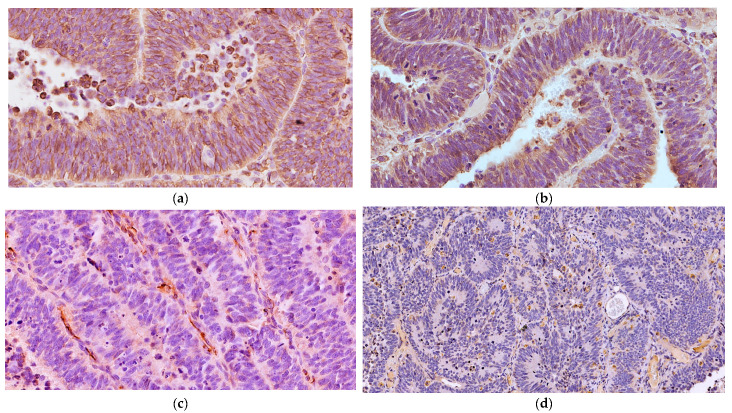
Spinal cord medulloepithelioma in the cat. (**a**) The tumor cells are positive for vimentin with a cytoplasmic and membranous distribution (VIM IHC, 400×). (**b**) The neoplastic cells are multifocally immunoreactive for NSE (NSE IHC, 400×). (**c**) The neoplastic cells show negative immunolabeling for GFAP with only some immunoreactive stromal cells (GFAP IHC, 400×). (**d**) The multilayered Homer Wright rosettes do not show immunolabeling with Olig2 (Olig2 IHC, 200×).

Microscopically, the neoplastic cells were densely packed, arranged as pseudostratified or multilayered trabecular and tubular structures (Figure 3a), supported by delicate vascular stroma with a PAS-positive external limiting membrane. The internal surface was covered with indistinct amorphous material, and limiting membranes were barely observed (Figure 3b). Some multilayered Homer Wright rosettes (i.e., an arrangement of cells surrounding an indistinct lumen) were occasionally present. In areas adjacent to trabecular structures, sheets of poorly differentiated cells were also found (Figure 3c). The cells had small, slightly elongated, and hyperchromatic nuclei with scant cytoplasm. Numerous mitoses (up to 30 mitoses per high power field, 400×, 0.237 mm^2^) were mainly observed at the luminal aspects of tubular structures, and the Ki-67 nuclear labeling index was up to 90% (Figure 3d). The stroma was composed of capillary structures, supported by sparse GFAP-positive cells. All neoplastic neuroepithelial cells were diffusely immunoreactive for vimentin (Figure 4a) and NSE (Figure 4b) and negative for GFAP (Figure 4c), Olig2 (Figure 4d), synaptophysin, PCK, S-100, NeuN, and nestin. Based on the histopathological and immunohistochemical results, a final diagnosis of MEPL was made.

## 3. Discussion

The morphological characteristics of the tumor were consistent with the diagnosis of MEPL due to the arrangement of folds and the irregular trabecular structures of the neoplastic pseudostratified neuroepithelium, reminiscent of the developing embryonic neural tube tissue with numerous mitoses.

Our immunohistochemical results revealed remarkable concordance with those of most human MEPLs, showing moderate to strong cytoplasmic and cell surface immunoreactivity for VIM and NSE and frequent nuclear immunolabeling for Ki-67 [28,29,31]. Similarly, the immunohistochemical staining for NSE was positive in an intraocular MEPL in a cat [10], as well as in those from a llama [12] and horses [1,4]. In contrast with the previously reported intraocular MEPL in cats [11], where GFAP was positive and VIM was negative in two cases, our results demonstrated GFAP negativity and VIM positivity, mirroring the findings in its llama [12], dog [8], horse [1,4], and human counterparts [23,28]. The immunostaining pattern of GFAP has yielded inconsistent results across various studies, with both positive and negative immunolabeling of MEPLs within the same species. Notably, S-100 immunostaining was negative in our study, similar to the results obtained in feline and canine MEPL [8,10].

In some ultrastructural studies of MEPL [27], the findings suggest that the external limiting membrane is distinct, resembling a basement membrane resting on a delicate connective tissue network. Contrastingly, the internal surface of the neuroepithelia exhibits an irregular amorphous coating with no identifiable structures of a basement membrane. Consequently, the presence of a PAS-positive internal limiting membrane is not considered a prerequisite for diagnosing MEPL.

In both human and veterinary medicine, MEPL is most frequently reported as an intraocular tumor, and two histological subtypes have been recognized. The teratoid subtype contains heteroplastic elements, such as cartilage, rhabdomyoblasts, and brain-like tissue, whereas the non-teratoid subtype does not contain heteroplastic elements and is composed of cells that resemble embryonic retinal cells. No such morphological distinction has been considered for the CNS counterpart in human pathology [32], although only one case of teratoid intraspinal MEPL has been reported [27]. The present case was classified as non-teratoid due to the absence of heteroplastic elements.

Spinal MEPL has been described in four young dogs as thoracolumbar intradural-extramedullary tumors: three dogs were 6 months old and one was 3 years old [20,21,22]. No cases of primary extra-ocular MEPL have been reported in other animals. In all described canine MEPL cases, histological descriptions of the tumors included the presence of tubules and acini lined by low columnar and pseudostratified epithelial cells. Based on such descriptions, it is actually believed that they were likely linked to ectopic nephroblastoma, thus indicating that the diagnosis of MEPL was a misdiagnosis [33].

Differential diagnoses of MEPL include ependymoblastoma, spinal cord nephroblastoma, intraspinal primitive neuroectodermal tumor (PNET), and embryonal tumor with abundant neuropil and true rosettes (ETANTR). In our case, neoplastic cells were consistently negative for GFAP, and there were neither true multilayered ependymoblastic rosettes to suggest ependymoblastoma [25] nor primitive glomeruli, Bowman’s spaces, or cytokeratin-immunolabeled components to suggest ectopic nephroblastoma [34]. Intraspinal PNET has been reported in a cat and was characterized by densely packed, undifferentiated small round cells [35]. The diagnosis of ETANTR, previously described in a cat with infratentorial localization [36], was excluded due to the absence of multilayered ependymoblastic true rosettes, neuropil-like areas, and foci of neurocytic differentiation.

In humans, spinal MEPLs have been exceptionally reported in two children [28,29] and one adult [27]. Extracranial and extraocular localizations (peripheral MEPLs) are also exceedingly rare. The discovery of a characteristic molecular alteration (i.e., amplification and fusions of the C19MC locus on chromosome 19q13.42) in tumors previously classified as ependymoblastoma, ETANTR, and a subset of CNS MEPLs has led to the current unified diagnostic term: “embryonal tumor with multilayered rosettes (ETMR), C19MC-altered” [37]. About 5% of human patients with intraocular MEPL have a DICER1 mutation, and intracranial MEPL in children has also been reported in the context of DICER1 syndrome [38]. So far, no genetic alterations have been investigated in such animal tumors, and consequently, we suggest that the adoption of the diagnostic term “medulloepithelioma” in veterinary pathology is still preferable when the histomorphology of the tumor resembles the embryonic neural tube.

In human medicine, the radiological features of MEPL are not specific; studies have shown isointensity or hypointensity in T1-weighted imaging and hyperintensity in T2-weighted imaging with T1-weighted imaging showing marked enhancement after the use of contrast agents [23,24,28]. Those findings are consistent with the results in our case and those of the reported case of feline PNET, although other intradural tumors, such as meningioma, nerve sheath tumor, lymphoma, and metastasis, show the same radiological pattern [35].

Despite sharing similar morphology, human intraocular MEPL demonstrates a slightly better prognosis compared to intracranial localization. However, there is insufficient information regarding the survival and prognosis of spinal MEPL [27]. Possible treatment options include surgery associated with radiotherapy and chemotherapy, especially in advanced stages with locoregional metastases. However, systemic chemotherapy is relatively unexplored with some controversy regarding its indication and efficacy. A protocol with vincristine, carboplatin, and etoposide proved to be an effective measure to prevent the recurrence and metastasis of advanced ocular MEPL [39].

In the cat in our report, the outcome immediately after surgery was excellent and remained stable for 14 months, but the recurrence of the neoplasm demonstrated its infiltrative nature. As this is the only reported case of feline spinal MEPL, additional cases are needed to consider the outcome and survival of this condition after surgery and possible radiotherapy and/or chemotherapy.

## 4. Conclusions

A spinal cord MEPL was diagnosed in a young cat with a temporarily favorable outcome after surgical removal. MEPL is an unusual tumor in cats, and cases of intraocular MEPL have been published in a few reports [10,11]. Considering the previous diagnoses of spinal MEPL reported in dogs being questionable, this is the first spinal cord MEPL report in animals, describing the clinical, neuroradiological, histological, and immunohistochemical findings. The inclusion of this tumor type within the WHO classification of primary tumors of feline CNS is suggested.

## Data Availability

All study data are presented in the article.
